# Retained free energy as a driving force for phase transformation during rapid solidification of stainless steel alloys in microgravity

**DOI:** 10.1038/s41526-018-0056-x

**Published:** 2018-11-19

**Authors:** Douglas M. Matson

**Affiliations:** Department of Mechanical Engineering, Tufts University, Medford, MA 02155 USA

## Abstract

Ternary Fe-Cr-Ni stainless steel alloys often exhibit a multi-step transformation known as double recalescence where primary ferrite converts to austenite during rapid solidification processes such as casting and welding. In addition to the volume free energy associated with undercooling between the phases, the free energy driving the transformation comes from two additional sources that are retained within the metastable solid—one from the primary phase undercooling and one from melt shear. A new physical model is proposed based on accumulation of defects, such as dislocations or tilt boundaries, and lattice strain. A dimensionless analysis technique shows that the free energy associated with metastable solidification is conserved and the contribution from melt shear can be predicted based on a modification of the Read-Shockley dislocation energy equation. With these additional terms the incubation time between nucleation events becomes inversely proportional to the total free energy squared for bulk diffusion and cubed for grain boundary diffusion mechanisms. In the case of the ferrous alloys studied, the grain boundary mechanism provides a better fit and when the model is applied the delay time behavior collapses to a single master-curve for the entire alloy family.

## Alloys: Tracking the evolution from liquid to solid

A model that describes the atomic-structure changes in a steel alloy as it rapidly changes from a molten to a solid state is developed by a scientist in the USA. Steel solidifies very quickly after being welded, during which process the atomic structure of the solid alloy can change multiple times. For example, an alloy of iron, chromium and nickel transforms from a ferrite atomic structure to one known as austenite. Classical nucleation theory does not explain this so-called double recalescence. Douglas Matson at Tufts University has developed a novel physical model to describe this microstructural evolution that is based on viewing the transformation as an accumulation of defects. The model predicts the time delay of the transformation, which Matson compares to observed values determined by microgravity experiments.

## Introduction

Molten metal processes such as casting or welding are commercially important manufacturing operations with casting shipments alone accounting for over $198 trillion/year worldwide.^1^ The US Materials Genome^2^ has identified improving modeling capabilities as an industry priority in order to significantly reduce product development time. Investigating process improvement options using electronic means through varying key parameters virtually, instead of conducting expensive trials, allows industry to be flexible in adapting to a changing market while increasing quality and profitability. Modeling increases speed to market, reduces development costs and allows the user to easily investigate company strategic position. Current software has matured to the point where mold filling, simultaneous fluid flow and heat transfer, local melt, mold, and solid thermal profile mapping, solidification, pore formation, trapped gas venting, interface tracking, and weld pool dynamics may all be successfully modelled. The next generation of models will include phase selection, localized solidification stress relaxation, and microstructural evolution for defect control. A missing piece of the puzzle is understanding how convection, through melt shear, influences microstructural evolution and this paper concentrates on developing a model to describe transformation kinetics in an important class of structural materials by looking at control of phase selection in Fe-Cr-Ni alloys during rapid solidification.

A key factor controlling nucleation and subsequent growth of solid phases is the undercooling relative to the melt. When a liquid is cooled to below its equilibrium melting temperature, solid forms and the heat of fusion from the growing crystal is rejected into the remaining liquid and growth only stops when the liquid reaches the melting temperature of the solid. This phenomenon also occurs when a second solid grows into a mixture of liquid and metastable solid in a process known as double recalescence. For alloys in this study the stable phase melts at a higher temperature than the metastable phase and thus the metastable undercooling is less than the stable-phase undercooling for a given melt temperature.

Local phase selection is thus based on a competition between nucleation and growth^3^ and the delay between nucleation of the primary metastable solid and the second stable solid is called the incubation time for the transformation. For ferrous alloys, the primary phase is often ferrite which subsequently transforms to austenite. Koseki^4^ showed that the nucleation of the second phase occurs preferentially at pre-existing grain boundaries within the metastable primary dendrites and the cluster geometry is characterized as two hemispherical caps.^5^ The physical mechanism requires grain boundaries that become potent when a critical number of dislocations become arrayed such that the surface energy promotes nucleation of the second phase.^6^

Classical nucleation theory^7,8^ is based on achieving a condition where addition of an atom to the cluster results in an overall decrease of free energy in the system. Volume free energy change represents a driving force which promotes nucleation while surface free energy involves an energy penalty which resists cluster formation as discussed in the supplemental material accompanying this article. The cluster becomes stable when it reaches a critical size sufficient to overcome the surface to volume ratio penalty in part based on the geometry of the nucleus. A key factor in this balance is the undercooling of the parent phase relative to the melting point of the crystalline solid which forms. The higher the undercooling, the greater the driving force for the transformation, and the shorter the incubation time. Here the subscript *s* represents conditions relative to the stable phase.

1 $$\Delta G_s = \frac{{\Delta H_s\Delta T_s}}{{T_s}}$$

Quantification of the transformation delay is based on the principle of microscopic reversibility, or principle of time reversal such that “fluctuations arise in the same manner as they decay”,^9^ where the rate at which a stable nucleus can form is equivalent to the rate at which it would decompose. Two key parameters for determining this rate are mass transfer from the surrounding parent phase and the attachment frequency of atoms onto the interface as they to join the cluster.^10–12^ Attachment is assumed to be thermally activated and is thus characterized by an Arrhenius relationship that describes the success rate for atoms joining the cluster. Herein we assume that cluster growth is some small fraction of the maximum interface flux due to diffusion; refer to supplementary material for additional details of this process. By mathematically eliminating the cluster critical radius using classical nucleation theory the delay τ becomes inversely related to the free energy change ΔG raised to a characteristic exponent which is geometry dependent. Not only is the geometry of the cluster important,^13^ but the mechanism for mass transfer must be identified.^14^ In this paper we will contrast a bulk diffusion attachment mechanism, where atoms join the cluster along both surfaces of the hemispherical cap ($\tau \propto \Delta G^{ - 2}$), with a grain boundary attachment mechanism, where atoms attach along the circular interface between cluster and the grain boundary ($\tau \propto \Delta G^{ - 3}$).

In either case, the experimentally measured delay times obtained by observing nucleation behavior are not explained by applying classical nucleation theory to Fe-Cr-Ni alloy double recalescence. For a given alloy we observe that the temperature following primary solidification is independent of undercooling because these systems do not partition appreciably, and metastable phase growth ends when the temperature rises to the ferrite melting point. Thus, the thermal driving force for any subsequent transformation from ferrite to austenite is constant Δ*T*_*s*_=*T*_*s*_−*T*_*m*_ and the incubation delay should be independent of undercooling or melt shear. In fact, the incubation time is a weak function of undercooling and a strong function of melt shear and the ramifications from these observations indicate that additional driving forces must be present since delays are significantly reduced. In the absence of partitioning, the remaining liquid is the same for all cases and thus the new theory involves identifying how the metastable phase must differ.

Microgravity testing is essential to understanding how melt shear due to stirring of the sample influences phase selection. Experiments on earth can only access the extreme conditions where either there is no convection or where there is highly turbulent flow. The transition from laminar to turbulent flow may only be investigated in microgravity and this work is based largely on analysis of experiments run using the ESA Electromagnetic Levitation Furnace (ISS-EML) on the International Space Station.^15^ Note that microgravity alone does not influence phase selection. Rather microgravity is enabling in order to conduct controlled experiments beyond what may be accomplished on earth.

## Results

### Retained damage model

**Fig. 1:**
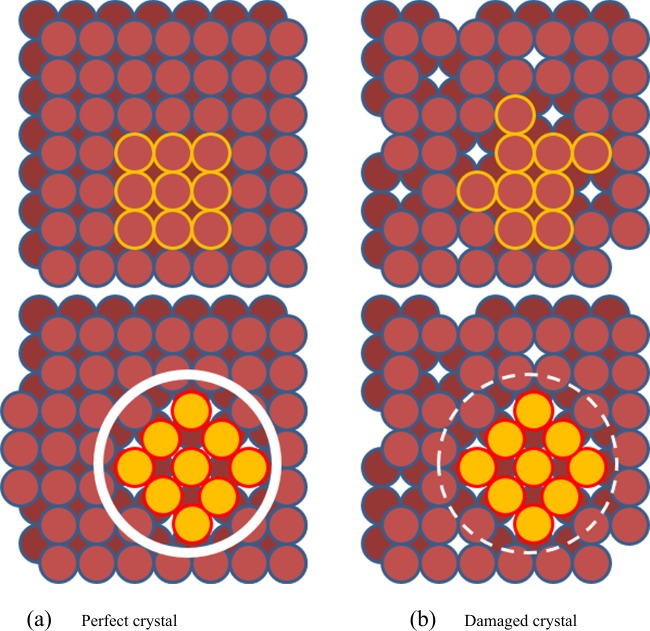
Schematic comparison of nucleation of a stable phase within a pre-existing metastable solid. **a** The primary phase has few defects or internal strain and significant mass transfer must occur to allow secondary nucleation, **b** the primary phase has many high-energy defects with associated internal strains—both conditions are relieved by secondary nucleation and the system energy decrease drives the transformation. In practice, this manifests itself as a reduction in the transformation delay

Since the incubation time is reduced with increased undercooling or melt shear the metastable solid must retain additional free energy to drive the transformation. Physically, we envision that this energy is embodied in an increase in microstructural defects that store energy which has been added to the crystal lattice during growth of the metastable phase or from damage to the lattice due to shear from melt convection. Figure 1 depicts a schematic of two crystal lattices which undergo the same transformation through nucleation of a critical cluster of second phase; the first represents a perfect crystal and the second a damaged crystal. In the first there is an energy penalty due to the formation of new surface by diffusion of parent atoms to the left side of the crystal. In the second, the pre-existing defects promote cluster formation through release of lattice strain without the need for surface creation.

Note that this overly simplified schematic depicts only point defects but real systems will most probably involve a combination of defects and retained damage including dislocation networks, tilt boundaries and lattice strain. Instead of attempting to discretize each contribution, a global energy approach is assumed. The key observations are that the more defect damage, the more retained energy in the metastable solid and the more retained stress, and thus the more energy available to drive the transformation. Either source can promote recrystallization or nucleation of new phases. By analogy to crystal growth processes the slower the crystal growth, through low undercooling, the fewer defects observed and the lower the melt shear during growth, through less stirring, the fewer the defects observed. Furthermore, by analogy to hot work, recrystallization of the parent phase may be an issue so it is advantageous to look at alloys exhibiting fast transformations where healing or dynamic recovery is minimized.

In evaluating experimental data using this model there are two independent measurements. The measured delay time is used to predict how much free energy was required to drive the transformation based on application of classical nucleation theory modified with the principle of microscopic reversibility. The measured pyrometry data is used to determine melt undercooling which independently defines how much free energy was available based on application of the retained damage model.

Test conditions for quiescent samples in the absence of induced flow are used to evaluate the influence of undercooling. The delay data is used to calculate how much free energy was required in to obtain the observed result; the pyrometry data is used to calculate how much free energy was available. The difference between these two results is the retained free energy. From these results the attachment mechanism can be defined by comparing the standard deviation for the model under each condition. Finally, test conditions for samples processed with different levels of induced stirring can be used to evaluate the influence of melt shear on the observed delay times. The pyrometry data is now used to define the sum of the free energy from secondary undercooling and retained free energy from primary undercooling. The difference between delay data predication and pyrometry data summation is now the retained shear free energy. The next two sections will concentrate on numerical evaluation of the contribution due to undercooling and melt shear, respectively.

### Influence of undercooling

For conditions where there is no melt convection any observed change in delay behavior must track back to differences retained from primary solidification. From the first law of thermodynamics, the limit to how much free energy may be retained Δ*G*_*m*_ is, by definition, the free energy associated with undercooling of the liquid with respect to the metastable solid melting temperature. Here the subscript m represents conditions relative to the metastable phase.

2 $$\Delta G_m = f_x\frac{{\Delta H_m\;\Delta T_m}}{{T_m}}$$

**Fig. 2:**
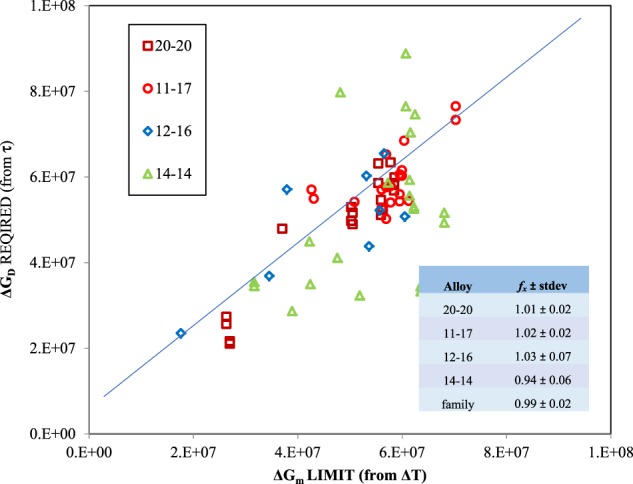
Conservation of free energy during primary solidification is demonstrated in a plot of free energy required to obtain the observed delay time as a function of the available free energy from metastable phase undercooling assuming a grain boundary attachment mechanism controls cluster growth. Tests were conducted under no-flow conditions using Electrostatic Levitation containerless processing. Four alloys (wt%) are shown, 60Fe-20Cr-20Ni (brown), 72Fe-11Cr-17Ni (red), 72Fe-12Cr-16Ni (blue), and 72Fe-14Cr-14Ni (green). The slope of the entire data set represents the fraction of metastable retained free energy *f*_*x*_ = 0.99 ± 0.02 with error based on the standard deviation (SD)

The retained free energy is some fraction of this available metastable volume free energy and a plot of the required free energy for the transformation Δ*G*_*D*_, from the observed delay, as a function of the limit to available free energy Δ*G*_*m*_, from the observed undercooling, will yield a slope equivalent to this fraction. This is shown in Fig. 2 for a variety of Fe-Cr-Ni alloy compositions within the stainless steel alloy family. The slope that is obtained approaches *f*_*x*_ = 1.0 within the statistical limits of the experimental data and the ratio of the standard deviation for a bulk mechanism is a factor of 1.5 higher than for a grain boundary mechanism indicating that grain boundary diffusion is likely the rate-controlling step for this alloy family. In the limit, free energy is conserved during growth of the metastable phase and is hereafter assumed to be retained to drive the subsequent transformation by augmenting volume free energy from undercooling relative to the stable phase Δ*G*_*s*_. Extrapolating this behavior to systems which exhibit changes in liquid composition due to partitioning makes it unlikely that free energy is fully conserved and for these alloys one would expect *f*_*x*_ *<* 1 unless solute trapping is sufficient to mitigate the implied irreversibility of the mechanism. Ferrous alloys tend to show very little partitioning and are particularly suited for evaluation of the new model.

### Influence of melt shear

**Fig. 3:**
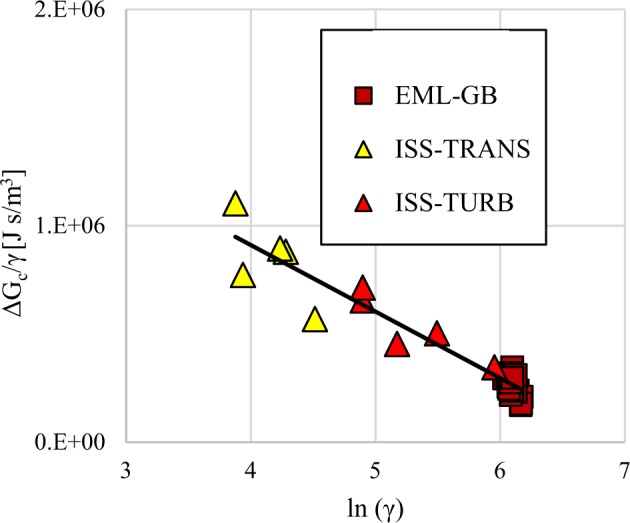
Evaluation of the empirical constants describing the retained damage free energy as a function of melt shear. Tests were run under transitional and turbulent conditions using Electromagnetic Levitation containerless processing. Ground-based tests (brown) were run with a single-frequency coil providing simultaneous levitation and heating while space testing on the International Space Station for turbulent transition (yellow) and fully-developed turbulence (red) conditions were run using a dual-frequency coil to decouple levitation and heating. Linear regression results in values for the intercept Δ_*b*_ = 2.140E6 ± 0.542E6 Js/m^2^ and slope Δ_*m*_ = 3.074E5 ± 0.177E5 Js/m^2^ which are used to predict the influence of convective melt shear on the incubation delay

During rapid solidification significant melt flow in the undercooled liquid only occurs prior to primary recalescence. Recalescence involves growth of dendrites across the sample such that a semi-solid mixture of finely dispersed metastable dendrites forms surrounded by liquid at the metastable solid melting point. Following primary recalescence the presence of this solid matrix limits the ability for convection, and the associated melt shear, to endure. As with the previous case where undercooling influences incubation time, any observed delay behavior change must track back to differences retained from primary solidification however now there exists no definitive thermodynamic limit to how much energy may be stored, although there is most likely an as-yet undefined physical limit. In developing an empirical approximation to the free energy associated with melt shear the retained damage model assumes a modified version of the Read-Shockley equation^16^ which describes the energy of a grain boundary as a function of the tilt angle formed due to regular spacing of dislocations along the boundary. In this model the number of dislocations along the boundary is inversely proportional to the spacing and proportional to the tilt angle. Thus, the higher the melt shear the greater the tilt angle. Although it is unlikely that retained energy is embodied solely by an array of edge dislocations, the form of the equation is used as a guide for approximation of real behavior. The model focuses on predicting the free energy that results from melt shear and is subsequently retained in the microstructure. Manipulation of the equation results in a linear relationship between a dependent variable $\Delta G_c/\dot \gamma$ and the independent variable $\dot \gamma$. This derivation is contained in the supplemental material associated with this article. Microgravity ISS-EML experimental data from tests run over a wide range of turbulent convective conditions yields an empirical estimation of the relationship between these two variables as shown in Fig. 3. Evaluation yields constants relating to the slope Δ_*m*_ and intercept Δ_*b*_ of the line which defines a predictive equation and allows calculation of the contribution to retained energy from convective melt shear as a function of the applied shear only. Although there is data available only for the 60Fe-20Cr-20Ni (wt%) system the empirical constants obtained by this analysis were assumed to apply to the entire family.

3 $$\Delta G_c = \Delta _m\;\dot \gamma \;\left[ {\Delta _b/\Delta _m - \;\ln \dot \gamma } \right]$$

The incubation delay may now be calculated for any given test condition. The delay is inversely proportional to the total free energy raised to the characteristic exponent and the total free energy has three terms corresponding to the constant term representing free energy due to sample undercooling relative to the stable phase, the test-specific free energy due to sample undercooling relative to the metastable phase and the test-specific free energy due to melt shear.

4 $$\Delta G_T = \Delta G_s + \Delta G_m + \Delta G_c$$

**Fig. 4:**
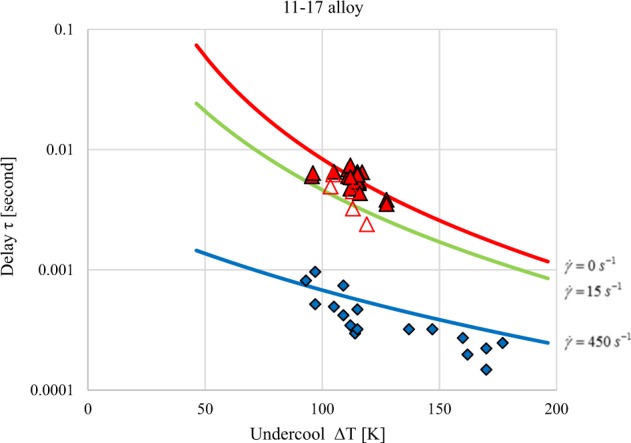
Experimental results are compared to model predictions for given melt shear conditions for the 72Fe-11Cr-17Ni (wt%) alloy. ESL (solid red) results are at no-flow conditions and thus there is no contribution to the delay driving force by convection and only undercooling influences the observed results. ESL with laser on (open red) is characterized by laminar flow due to Marangoni convection and thus approaches the low shear model predictions; again undercooling dominates. EML single-frequency testing (blue) is fully turbulent and results are shear dominated. Note that tests from a specific facility will not have constant shear because as undercooling increases, the melt viscosity will also increase significantly thus reducing recirculation velocities; melt shear is proportional to recirculation velocity

The predictions for three levels of melt shear representing no-flow, laminar and turbulent flow condition are shown in Fig. 4, where the experimentally observed delay times are plotted as a function of undercooling for the 72Fe-11Cr-17Ni (wt%) alloy. Samples tested under quiescent conditions show agreement with predictions for no-flow while samples tested with significant stirring show agreement with predictions for high shear as predicted by magnetohydrodynamic modeling of droplet convection for the test conditions specified.^17^

### Dimensional analysis

Double recalescence is only possible if the undercooling is great enough to access the metastable phase; in practice this requires the melt temperature to drop below the equilibrium melting point of the metastable ferrite. This minimum undercooling Δ*T*_*s*_ is taken as the baseline for developing a dimensionless approach and the reference delay time and reference driving force are evaluated at this temperature. The dimensionless delay time is thus the ratio of the observed delay for a given test to the reference value *N*_*τ*_=*τ*/*τ*_*R*_. Note that this reference value is the longest delay possible for any given alloy composition, but the value will vary depending on the thermophysical properties of the given alloy. The dimensionless driving force is the ratio of the predicted total free energy, including all retained energy due to both undercooling and convection, to the reference value *N*_M_=Δ*G*_*T*_/Δ*G*_*R*_. Note that this reference value Δ*G*_*R*_ *=* Δ*G*_*s*_ is the smallest driving force possible for any given alloy since Δ*G*_*m*_ *=* Δ*G*_*c*_ = 0 but the value will vary with composition due to differences in thermophysical properties for any specific composition. The interrelationship between the reference values is readily apparent given the observation that the longest delay results from the smallest driving force. Refer to supplemental information for additional details.

**Fig. 5:**
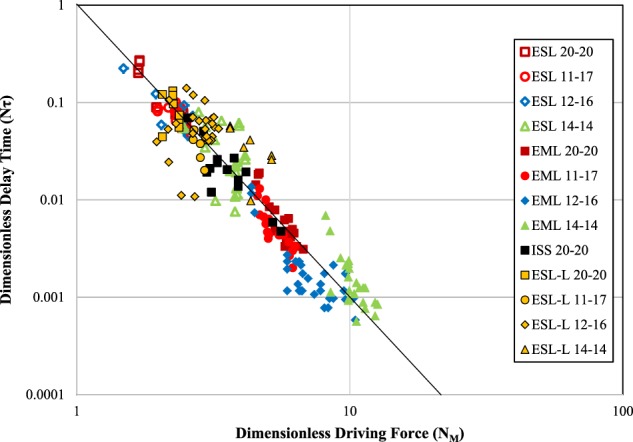
Implications of the retained damage free energy model showing the transformation delay as a function of driving force for Fe-Cr-Ni alloys processed over a wide range of convective conditions. Four alloys (wt%) are shown, 60Fe-20Cr-20Ni (brown), 72Fe-11Cr-17Ni (red), 72Fe-12Cr-16Ni (blue), and 72Fe-14Cr-14Ni (green); open symbols represent no-flow conditions in ESL, filled represent fully-turbulent conditions in single-frequency EML. ISS laminar-transitional-turbulent flows (black) and laminar Marangoni-flow ESL with laser on (yellow) are also included for comparison

By utilizing the dimensionless approach on a series of tests across a wide range of compositions within the Fe-Cr-Ni family, the results collapse onto a single master-curve as seen in Fig. 5. Linear regression for the log-log plot yields a slope of −2.98 ± 0.03 which compares favorably to the theoretical value of −3 as indicated by the line in the Figure. The coefficient of determination for the dataset is *R*^*2*^ = 0.89 when compared to theory indicating that a grain boundary mechanism controls attachment to the cluster and emphasizes the importance of locating the cluster along pre-existing grain boundaries.^4–6^

## Discussion

Experimental observations show that both primary undercooling and convection cause the incubation delay during ferrite–austenite transformation to be reduced in many ferrous alloy systems. From classical nucleation theory this transformation is known to be driven by changes in bulk free energy content; the higher the driving force the faster the transformation. The retained damage model is based on the assumption that the free energy of the microstructure is augmented by defect energy in a process analogous to that observed during hot working. This retained free energy is not specifically assigned to a given defect type but rather is interpreted as a global contribution. The augmentation due to primary undercooling is thermodynamically limited to the free energy that was available during primary undercooling. The augmentation due to convection can be quantified using a modified Read-Shockley approach which relates energy stored to defect concentration. Dimensional analysis for a given alloy is based on normalization of experimental results at conditions where the undercooling corresponds to the difference in temperature between the melting points of stable austenite and metastable ferrite and equivalent to the minimum undercooling where the metastable transformation can be accessed. Using the dimensionless approach the behavior of delay and driving force normalized to the baseline condition yields a single line across a broad composition range for alloys with very different thermophysical properties. Two key assumptions are required: the attachment mechanism for cluster growth is controlled by a grain boundary mass transfer process and attachment kinetics are reduced from the rate that diffusion could occur by some common empirically-defined family-specific attachment frequency success factor as discussed in the supplemental material.

Because the dimensionless approach line provides a universal fit to all data for the stainless steel alloy family, phase selection may now be included in modeling of welding and casting processes. Real systems tend to show low undercoolings and the delay time will be dominated by fluid shear effects. Thus, the overall impact of this work is that it provides a framework for predicting phase selection to control local variability within a specific dynamic melt environment. Note that only alloys with very short delay times and limited partitioning were included in this study and the potential effects of microstructural healing have not been investigated. Future work is needed to identify how dynamic recovery may influence the incubation time and if free energy may be retained long enough to enhance solid-phase transformations which may occur following completion of equilibrium solidification.

In conclusion, classical nucleation theory can be used to predict the ferrite-to-austenite transformation delay by invoking the principle of microscopic reversibility when undercooling is just below the cusp of accessing the metastable phase—this condition is selected as the reference condition for subsequent dimensional analyses. For all undercoolings higher than this limit the delay is inversely proportional to the total free energy raised to a characteristic exponent that is geometry and mass transfer dependent. The level of undercooling below this critical value provides the thermodynamic driving force that drives primary solidification during double recalescence. This driving force is conserved through being retained within the metastable solid and augments the thermodynamic driving force that drives secondary phase solidification. Melt shear adds additional retained free energy and an empirical technique based on the Read-Shockley dislocation energy theory is used to quantify this effect and although data is only available for one alloy the results are successfully applied to the entire alloy family. The total free energy driving the transformation is the sum of these three contributions and a plot of dimensionless delay to dimensionless driving force collapses to a single line for a wide variety of alloy systems with tests spanning a broad range of melt shear conditions, from laminar to turbulent flow, across multiple test facilities.

## Methods

Details on specimen fabrication and measurement techniques have been documented by Kensel.^18^ The alloys selected were 60Fe-20Cr-20Ni (20–20), 72Fe-11Cr-17Ni (11–17), 72Fe-12Cr-16Ni (12–16), and 72Fe-14Cr-14Ni (14–14) all in weight percent. Details on the microgravity sample are documented by ESA.^19^

All tests were conducted using containerless processing techniques to minimize the potential for melt contamination and suppress the effects from crucible wall heterogeneous nucleation. No-flow conditions were achieved using an electrostatic levitation facility (ESL) at NASA Marshall Space Flight Center^20^ where nominal 2 mm diameter (30–50 mg) samples are processed in vacuum to minimize arcing; samples are melted using a laser and radiatively cooled by turning the laser off. Laminar flow conditions were achieved by leaving the laser on (ESL-L) at a selected low power to induce Marangoni flow during cooling. Where the laser illuminates the sample the surface is hottest and on the opposing-side the surface is coldest. Differences in surface tension with temperature drive internal flow. Turbulent flow conditions were achieved using an electromagnetic levitation system (EML)^18^ where 6.5 mm diameter samples (1 g) are processed in argon at ambient pressure; following melting the samples are cooled convectively by blowing ambient pressure helium across the surface. The EML levitation coils use a single-frequency RF oscillating current to simultaneously position and heat the sample and gravity pulls the sample down into the coil inducing significant convection. In order to conduct tests spanning the range from laminar to turbulent flow regimes using a single facility it is desirable to remove the effects of gravity. A single sample of 60Fe-20Cr-20Ni (wt%) was processed in microgravity aboard the International Space Station using the ISS-EML electromagnetic levitation facility through a collaboration between NASA, ESA and the German Space Agency DLR-Köln.^15^ This facility uses a dual-frequency coil that superimposes two RF oscillating currents to independently control positioning and heating. The 6.5 mm diameter sample was processed in 450 mbar helium or argon to vary the conductive cooling rate.

Magnetohydrodynamic modeling^17^ is used to predict induced convection and melt shear as a function of melt properties, applied power, and thermal gradient. Note that melt shear is shown to vary linearly with local recirculation velocity and conditions vary significantly within a single sample; thus the reported values for velocity or shear are based on maximum values observed within the flow field.

Statistical analysis for reporting of variability and error are based on standard linear regression techniques with reporting of one-sigma deviation equivalent to 68% confidence intervals. The coefficient of determination evaluation of alloy family behavior is based on comparison of experimental results to theory using the log-log data presented in Fig. 5.

## Electronic supplementary material

Supplemental Material

## Data Availability

The data that support the findings of this study are available from the corresponding author upon reasonable request.

## Electronic supplementary material

**Supplementary information** accompanies the paper on the *npj Microgravity* website (10.1038/s41526-018-0056-x).
