# Supplementary material for: Retained free energy as a driving force for phase transformation during rapid solidification of stainless steel alloys in microgravity
Source: NPJ Microgravity. 2018 Nov 19;4:22. doi: 10.1038/s41526-018-0056-x (PMC6242990; doi:10.1038/s41526-018-0056-x)
Supplement: Supplementary file 1 — Supplemental Material [file 41526_2018_56_MOESM1_ESM.docx]

**Supplemental Materials**

**Table S1: Nomenclature**

__________________________________________________________________________

a_o_ (m) Lattice constant

A (m^2^) Area along an interface

C (- -) fractional solute concentration (for bcc steel this is fcc Ni)

D (m^2^/s) Diffusivity

D_o_ (m^2^/s) Arrhenius pre-exponential constant in diffusivity

E (J/m^2^) Read-Shockley energy of a grain boundary

f_B_ (- -) Attachment fraction as compared to diffusion

f_R_ (- -) Fraction or retained energy lost due to recovery processes

f_x_ (- -) Fraction of free energy retained

ΔG_c_ (J/m^3^) Retained free energy from convection

ΔG_D_ (J/m^3^) Retained free energy from observed delay, relative to the metastable phase

ΔG_m_ (J/m^3^) Retained free energy from undercooling, relative to the metastable phase

ΔG_R_ (J/m^3^) Reference free energy

ΔG_s_ (J/m^3^) Reference free energy relative to stable phase

ΔG_T_ (J/m^3^) Total free energy

ΔG_v_ (J/m^3^) Volume free energy from classical nucleation theory

ΔH (J/mol) Enthalpy of fusion

J (at/m^2^s) Atomic attachment flux

k_B_ (J/atK) Boltzmann constant

n (at) Number of atoms in a cluster

N_A_ (at/mol) Avagadro’s number

N_M_ (- -) Dimensionless driving force ratio

N_τ_ (- -) Dimensionless delay time ratio

N_Ξ_ (- -) Dimensionless thermophysical property ratio

Q (kJ/mol) Arrhenius activation energy in diffusivity

r (m) Radius, with * for critical radius

ΔS (J/molK) Entropy of fusion

t_o_ (s) Extrapolated incubation intercept

T_m_ (K) Melting temperature of metastable phase

T_s_ (K) Melting temperature of stable phase

ΔT (K) Experimentally observed undercooling

V (m^3^) Volume of cluster

*β* (at/s) Cluster atomic attachment rate

*δ* (m) Grain boundary thickness

Δ_b_ (Js/m^2^) empirical intercept constant relating shear and retained energy

Δ_m_ (Js/m^2^) empirical slope constant relating shear and retained energy

*γ* (J/m^2^) surface energy

 (s^-1^) shear rate

τ (s) Experimentally observed incubation delay

τ_R_ (s) Reference incubation delay

τ_EXP_ (s) Experimentally observed incubation delay

θ (radian) Wetting angle from classical nucleation theory

θ (radian) Read-Shockley tilt grain boundary angle due to an array of dislocations

κ (- -) Microstructural reversibility constant

Ω (m^3^/mol) Atomic volume

Subscripts

M Metastable phase

S Stable phase

* Critical cluster attribute

__________________________________________________________________________

**Supplementary Notes**

**Size of a critical nucleus from classical nucleation theory** REF [S1, S2]

$$\begin{matrix} \Delta G_{cap}=-V \Delta G_{v}+A_{S} \gamma_{M/S}-A_{M} \gamma_{M/M} \left.\frac{\partial\left(\Delta G_{cap}\right)}{\partial r}\right|_{critical}=0 \\ r*=\frac{2 \gamma_{M/S}}{\Delta G_{v}} \Delta G*=\frac{16 \pi}{3}\frac{\gamma_{M/S}^{3}}{\Delta G_{v}^{2}}f(\theta) \end{matrix}$$

$$\begin{matrix} \cos\theta=\frac{\gamma_{M/M}}{2 \gamma_{M/S}} f\left(\theta\right)=\frac{1}{2} \left(1-\cos\theta\right)^{2} \left(2+\cos\theta\right) \\ V=\frac{4}{3} \pi r^{3} f\left(\theta\right) \\ A_{S}=4 \pi r^{2} \left(1-\cos\theta\right) \\ A_{M}= \pi r^{2} \left(1-\cos^{2}\theta\right) \\ n*=\frac{32 \pi}{3} \frac{\gamma_{M/S}^{3}}{\Delta G_{V}^{3}} \frac{N_{A}}{\Omega_{\gamma}} f\left(\theta\right) \end{matrix}$$

**Evaluation of volume free energy from undercooling from classical nucleation theory** - REF [S3]

Assume entropy is temperature independent and evaluated at the melting temperature. Apply this value to any arbitrary temperature to obtain a function of volume free energy as a function of undercooling

$$\begin{matrix} \left.\Delta G_{v}\right|_{T_{L}}=\Delta H-T_{L} \Delta S=0 \\ \Delta G_{v}=\Delta H-T \left(\frac{\Delta H}{T_{L}}\right)=\Delta T\frac{\Delta H}{T_{L}} \end{matrix}$$

**Principle of microstructural reversibility** REF [S4]

$\tau=-\frac{\kappa k_{B} T}{\beta\left(\frac{\partial^{2}\Delta G}{\partial n^{2}}\right)}$ The negative term arises because $\frac{\partial^{2}\Delta G}{\partial n^{2}}<0$

0.5 < κ < 5 for solid-fluid interfaces and substrates with surface diffusion REF [S5].

κ = 4 for gas condensation REF [S4], and for condensed phases REF [S6] and for GB interfaces REF [S7] – thus it is appropriate to use κ = 4.

$$\begin{matrix} \Delta G_{cap}=-V \Delta G_{v}+A_{S} \gamma_{M/S}-A_{M} \gamma_{M/M} \\ V=\frac{4}{3} \pi r^{3} f\left(\theta\right)=\frac{n \Omega}{N_{A}} n=\frac{4}{3} \pi r^{3} f\left(\theta\right)\frac{N_{A}}{\Omega} \frac{dn}{dr}=\frac{8}{3} \pi r^{2} f\left(\theta\right)\frac{N_{A}}{\Omega} \\ A_{S}=4 \pi r^{2} \left(1-\cos\theta\right) A_{M}= \pi r^{2} \left(1-\cos^{2}\theta\right) \\ \frac{\partial\Delta G}{\partial n}=\frac{\partial\Delta G}{\partial r}/\frac{\partial n}{\partial r}=\frac{\Omega_{S}}{N_{A}} \left(\frac{2\gamma_{M/S}}{r}-\Delta G_{v}\right) \frac{d}{dr}\left(\frac{\partial\Delta G}{\partial n}\right)=\frac{\Omega_{S}}{N_{A}} \left(-\frac{2\gamma_{M/S}}{r^{2}}\right) \\ \frac{\partial^{2}\Delta G}{\partial n^{2}}=\frac{d}{dr}\left(\frac{\partial\Delta G}{\partial n}\right)/\frac{\partial n}{\partial r}=-\frac{\gamma_{M/S}}{2 \pi r^{4}f\left(\theta\right)}\frac{\Omega_{S}^{2}}{N_{A}^{2}} \left.\frac{\partial^{2}\Delta G}{\partial n^{2}}\right|_{r*}=-\frac{\Delta G_{v}^{4}}{32 \pi\gamma_{M/S}^{3}f\left(\theta\right)}\frac{\Omega_{S}^{2}}{N_{A}^{2}} \end{matrix}$$

Attachement flux $J=\frac{\beta}{A_{s}}=f_{B} \left(\frac{D}{a_{o}^{2}}\right)\left(C a_{o} \frac{N_{A}}{\Omega}\right)$ REF [S8]

Where the attachment rate is some fraction *f_B_*·of the diffusion rate *D*

Combining for each attachment mechanism yields

BULK $A_{s}=4 \pi r^{2} \left(1-\cos\theta\right)$ $\tau=\frac{4 k T_{LB} a_{o} N_{A} \gamma_{M/S}\left(1-\cos\theta\right)\left(2+\cos\theta\right)}{\Delta G_{v}^{2} C f_{B-BULK} D_{BULK} }\left(\frac{\Omega_{B}}{\Omega_{F}^{2}}\right)$

GB $A_{s}=\delta2 \pi r \sin\theta$ $\tau=\frac{16 k T_{LB} a_{o} N_{A} \gamma_{M/S}^{2}}{\Delta G_{v}^{3} C f_{B-GB} \delta D_{GB} }\frac{\left(1-\cos\theta\right)^{2}\left(2+\cos\theta\right)}{\sin\theta}\left(\frac{\Omega_{B}}{\Omega_{F}^{2}}\right)$

**Read-Shockley theory modified for application to melt shear** REF [S9]

Slope and intercept evaluation for 60Fe-20Cr-20Ni (wt%) shown in **Figure 3**

**Dimensionless groups defined by alloy-specific reference condition**

Free energy $N_{M}=\frac{\Delta G_{T}}{\Delta G_{R}}$ with $\Delta G_{R}=\frac{\Delta H_{s} \Delta T_{s} }{T_{s} }$

Delay $N_{\tau}=\frac{\tau_{EXP}}{\tau_{R}}$

Special case when thermophysical properties of stable and metastable alloy are similar

$$N_{M}=\frac{\Delta G_{T}}{\Delta G_{R}}=\frac{\Delta G_{R}+\Delta G_{D}}{\Delta G_{R}}=1+\frac{\Delta G_{D}}{\Delta G_{R}}$$

$$N_{\Xi}=\frac{\Delta H_{M} T_{M}/\Omega_{M}}{\Delta H_{S} T_{S}/\Omega_{S}} \frac{\Delta G_{T}}{\Delta G_{R}}=1+\left(\frac{\Delta T}{\Delta T_{s}}-1\right) N_{\Xi}$$

$$if N_{\Xi}\to1 then \frac{\Delta G_{T}}{\Delta G_{R}}\to\frac{\Delta T}{\Delta T_{s}}$$

**Table S2: Thermophysical properties common to all compositions**


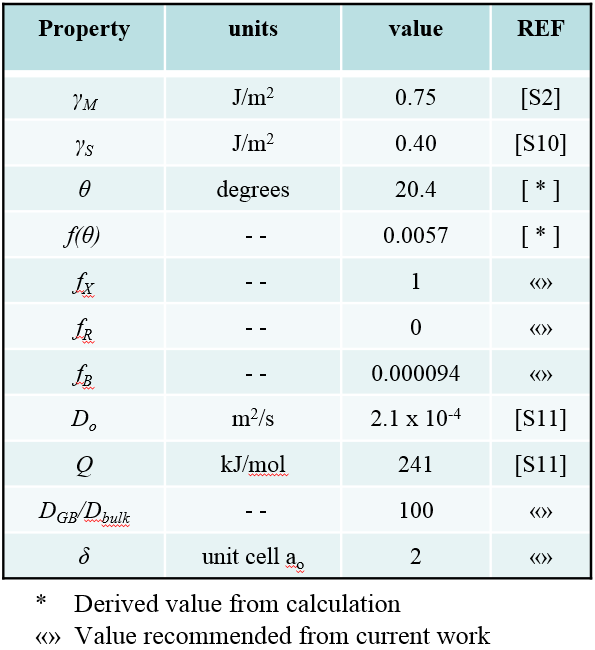


Note that Kantrowicz REF [S12] used a value of *f_B_* = 5x10^-4^ for nucleation of water from vapor.

**Table S3: Properties specific to each alloy**


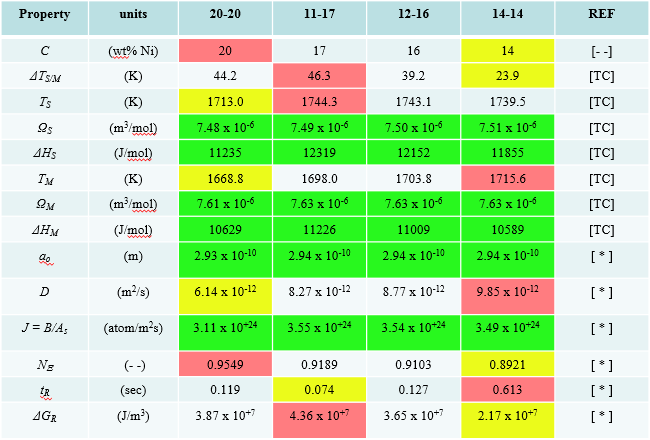


Reference column includes values calculated using ThermoCalc [TC] or derived by calculation [*]

Values are color-coded to emphasize where values are similar for all compositions (green) or where there is significant deviation high (red) and low (yellow).

**Supplementary References**

[S1] J.W. Christian, The Theory of Transformation in Metals and Alloys, Pergamon Press, Oxford, 418-475 (1975).

[S2] D. M. Matson and X. Xiao, "Identifying metastable interface potency limits during steel alloy transformations", *Materials Letters* **212**, 256-258 (2018).

[S3] D. Turnbull, “Formation of Crystal Nuclei in Liquid Metals”, *J. Appl. Phys*. **21**, 1022-1028 (1950).

[S4] J. Feder, K. C. Russell, J. Lothe and G. M. Pound, “Homogeneous nucleation and growth of droplets in vapors”, *Advan. Phys.* **15**[57], 111-178 (1966).

[S5] S. Toschev and I. Gutzow, “Time lag in heterogeneous nucleation due to nonstationary effects”, *Phys. Status Solidi* **21**[2], 683-691 (1967).

[S6] K. C. Russell, “Linked flux analysis of nucleation in condensed phases”, *Acta Met.* **16**, 761-769 (1968).

[S7] K. C. Russell, “Grain boundary nucleation kinetics”, *Acta Met.* **17**, 1123-1131 (1969).

[S8] G. Shao and P. Tsakiropoulos, “Prediction of phase selection in rapid solidification using time dependent nucleation theory”, *Acta Metall. Mater*. **49** [9], 2937-2942 (1994).

[S9] W. T. Read and W. Shockley, “Dislocation models of crystal grain boundaries”, *Phys. Rev.* **78**, 275-289 (1950).

[S10] T. Koseki and M.C. Flemings, “Solidification behavior of undercooled Fe-Cr-Ni alloys: Part I. thermal behavior”, *Met. Trans.* **26**A, 2991-2999 (1995).

[S11] D. W. James and G. M. Leak, “Self-diffusion and diffusion of cobalt in alpha and delta-iron”, *Phil. Mag*. **14:130**, 701-713 (1966).

[S12] A. Kantrowictz, “Nucleation in very rapid vapor expansions”, J. Chem. Phys. **19**, 1097-1100 (1951).
